# Does a Warmer World Mean a Greener World? Not Likely!

**DOI:** 10.1371/journal.pbio.1002166

**Published:** 2015-06-10

**Authors:** Jonathan Chase

**Affiliations:** Freelance Science Writer, Leipzig, Germany

Despite the “gloom and doom” scenarios depicted by most climate change scientists, the warmer world that we are creating can’t be all bad, can it? After all, hundreds of studies have shown that plant productivity is higher when temperatures are warmer and atmospheric carbon dioxide is high. Many models even predict an increase in global plant productivity, which has provided fodder for many to advocate the benefits of climate change to humans. Why wouldn’t we want a warmer and CO_2_-enriched world if it means higher productivity of plants, especially in impoverished regions where even slight increases in plant production could mean the difference between starvation and prosperity?

Unfortunately, the simple idea that global warming could provide at least some benefits to humanity by increasing plant production is complicated by a number of factors. It is true that fertilizing plants with CO_2_ and giving them warmer temperatures increases growth under some conditions, but there are trade-offs. While global warming can increase plant growth in areas that are near the lower limits of temperature (e.g., large swaths of Canada and Russia), it can make it too hot for plant growth in areas that are near their upper limits (e.g., the tropics). In addition, plant productivity is determined by many things (e.g., sunlight, temperature, nutrients, and precipitation), several of which are influenced by climate change and interact with one another. And so while we have hundreds of models that are available to project future climate change based on a number of different scenarios, we really have little clue as to what this future climate-changed world might look like in terms of plant production—that is, until now.

In this issue of *PLOS Biology*, Mora and colleagues provide a broader view of how global warming will likely change plant productivity in several ways. First, they include multiple factors that influence plant growth and are expected to be affected by climate change—temperature, soil water content, and sunlight levels –and their interactions. Second, they examine expected changes in different regions across the globe, examining the net increases and decreases in the number of days suitable for plant growth under climate change. Third, they examine the correlations between these expected changes in different regions and the vulnerability of people living in those regions. The answers they find, in a nutshell, are that if carbon emissions remain on their current trajectory (the status quo), the losses of plant production are likely to be far greater than any gains when examined at a global scale, and a substantial number of people, especially those who are most impoverished, will be at greater risk. Importantly, however, Mora and colleagues repeated their analysis for several global change scenarios and found that these changes will be much more moderate if society agrees to restrict carbon emissions in real, but manageable, ways.

**Fig 1:**
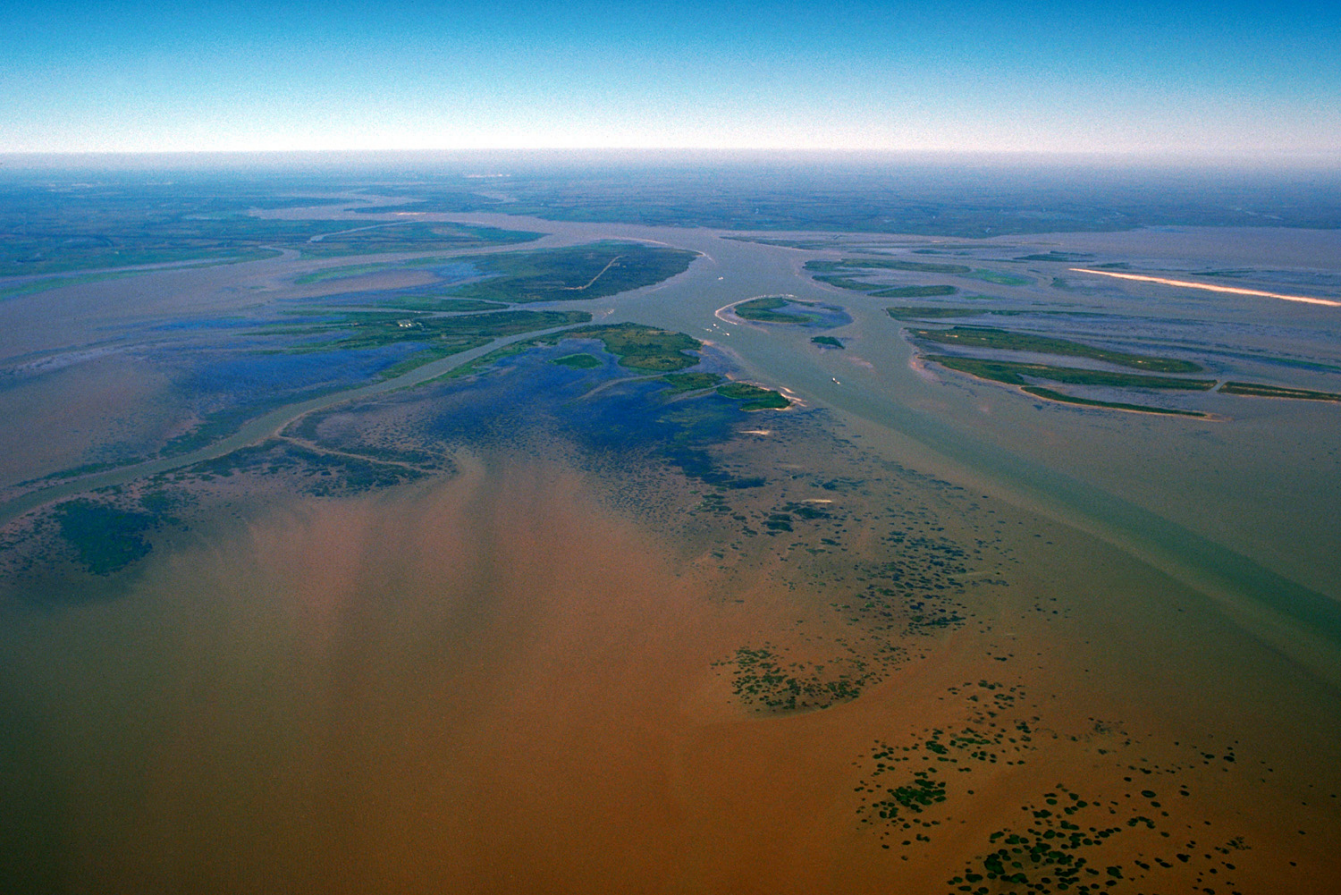
Many terrestrial ecosystems are vulnerable to losses in plant primary production, because of reductions in the number of days when climatic variables are suitable for plant growth. Higher latitudes will gain suitable climate days because of warming, but such gains cannot be fully capitalized because of lack of solar radiation. In turn, tropical ecosystems will lose climate days because of warming and drought. *Image credit*: *Arthur Belala*, *United States Army Corps of Engineers*

A look into the nitty-gritty of Mora and colleagues’ analyses shines some light on how they arrived at these conclusions. First, they used remote sensing satellite data to correlate how plant growth responded to varying levels of temperature, soil moisture, and solar radiation. From this, they estimated the range of climate conditions that enabled plant growth on our planet. These thresholds then allow them to calculate the numbers of days in a given year in which positive plant growth was expected in each region of the world. Next, they used daily projections of Earth Systems Models into the year 2100 for temperature, soil moisture, and radiation in regions across the world in order to simulate changes in the number of suitable plant growing days. They compared plant growth days from today to those under three climate change scenarios, including the status quo on carbon emissions, which would lead to more than a doubling of current amounts of atmospheric CO_2_ (~930 ppm) by 2100, and two scenarios in which emissions levels are actively reduced. If emissions remain unchecked, the analysis by Mora and colleagues shows that the number of days with climatic conditions suitable for plant growth would only increase in a few places (China, Russia, and Canada), whereas many other places, particularly in the tropics, will show dramatic declines, leading to an overall decline in plant growing days across the globe by more than 10% (Fig 1). Importantly, however, even if moderate controls on emissions are accomplished by society, this projected loss of plant growing days all but disappears.

The second main thrust of Mora and colleagues’ analysis was to link their predicted changes in plant growth days in different places across the world to the human populations that live in those places. In tropical evergreen forests, for example, a status-quo scenario predicts that plants will lose up to 25% of their suitable growing days because of temperatures that are too warm. A huge number of people in these regions depend on plants (both from forests and agriculture) for food, fiber, and fuel, and many of these people are also impoverished; they have little means to adapt if these goods and services are impaired by climate change. Mora and colleagues examined how their projected estimates of changes in the numbers of plant growing days correlated with two aspects of the human populations exposed to these changes: dependency on plant resources and their social adaptability to change. They found that under the status-quo scenario, nearly 3.5 billion people could be exposed to reductions of plant growth days by 30% or more. Of those, nearly 3 billion are highly dependent on plant resources, and 2 billion are also in low-income countries that are likely to suffer the most from those changes. On the other side of the coin, only 270 million or so people live in countries that are projected to experience significant increases in plant growing days (e.g., Scandinavia).

Mora and colleagues’ analysis contradicts the currently assumed “silver lining” of global warming—that, despite the many documented costs of global warming, at least plant growth will be enhanced. Instead, there are likely to be many more parts of the world that experience reduced, rather than increased, plant growth as a result of global warming, and this will likely have a negative impact on a large proportion of the world’s population. There is, however, a different silver lining in Mora and colleagues’ analysis. If our global society is able to come together and restrict emissions even a moderate amount, the magnitude of this predicted change in global plant productivity and human well-being will be substantially reduced.
